# From Structure to Sensing: Molecular Mechanistic Insights into Plant-Derived Carbon Dots for Heavy Metal Ion Detection

**DOI:** 10.3390/nano14211766

**Published:** 2024-11-03

**Authors:** Himanshi Soni, Vicky Jain, Suhas Ballal, Indang Ariati Ariffin, Mamata Chahar, Suman Saini, Monika Bhattu, Harbinder Singh, Mikhael Bechelany, Jagpreet Singh

**Affiliations:** 1 Centre of Research Impact and Outcome, Chitkara University, Rajpura 140417, Punjab, India; shimanshi543@gmail.com; 2 Marwadi University Research Center, Department of Chemistry, Faculty of Science, Marwadi University, Rajkot 360003, Gujarat, India; vicky.jain@marwadieducation.edu.in; 3 Department of Chemistry and Biochemistry, School of Sciences, JAIN (Deemed to be University), Bangalore 560027, Karnataka, India; b.suhas@jainuniversity.ac.in; 4 Management and Science University, Shah Alam 40100, Selangor, Malaysia; research5@msu.edu.my; 5 Department of Chemistry, NIMS Institute of Engineering & Technology, NIMS University Rajasthan, Jaipur 303121, Rajasthan, India; mamata.chahar1@nimsuniversity.org; 6 Department of Applied Sciences, Chandigarh Engineering College, Chandigarh Group of Colleges—Jhanjeri, Mohali 140307, Punjab, India; suman499.research@cgcjhanjeri.in; 7 Research & Incubation Centre, Department of Chemistry, Rayat Bahra University, Mohali 140103, Punjab, India; bhattumonika@gmail.com; 8 Parul Institute of Applied Sciences, Parul University, Vadodara 391760, Gujarat, India; 9 University Centre for Research and Development, Chandigarh University, Mohali 140413, Punjab, India; harvinder90@gmail.com; 10 Institut Européen des Membranes, IEM, UMR-5635, University of Montpellier, ENSCM, CNRS, Place Eugène Bataillon, 34095 Montpellier, France; 11 Functional Materials Group, Gulf University for Science and Technology (GUST), Mubarak Al-Abdullah 32093, Kuwait

**Keywords:** carbon dots, environment, heavy metal, natural precursors, toxicity, quenching

## Abstract

Plant-derived carbon dots (P-CDs) are gaining attention in environmental remediation due to their cost-effectiveness, availability, and lower toxicity compared with chemically synthesized carbon dots. This review comprehensively examines the recent advancements in the synthesis and application of P-CDs, with a particular emphasis on their efficacy in the sensing of heavy metals, which are among the most pervasive environmental contaminants. A detailed comparative analysis is presented by evaluating the performance of P-CDs against their chemically synthesized counterparts based on key parameters, such as optimal operating conditions and detection limits. Furthermore, sensing the potential of P-CDs towards every heavy metal ion has been discussed with in-depth mechanistic insights. Additionally, this review explores the industrial applications and future directions of P-CDs. This review provides a comprehensive analysis of -P-CDs for heavy metal sensing, aiming to enhance their sensitivity and selectivity toward heavy metal ions.

## 1. Introduction

In today’s world, there is an increasing demand for addressing environmental concerns, making sustainable development a crucial area of research [1]. One of the major issues faced by today’s society is the sudden increase in heavy metals contamination, and the reasons include industrialization, urbanization, pharmaceutical waste, and the use of harmful pesticides. Heavy metal ions are receiving much attention owing to their increasing adverse effects on mankind and the environment [2]. Due to the intensive growth of crops, a large number of pesticides and insecticides are used in agriculture that contain heavy metals in their formulations. Additionally, industrial discharges of heavy sludge each year contain traces of the aforementioned contaminants. Among the various metals, lead, mercury, and aluminum are considered the most harmful elements and pose severe health issues even at low concentrations. Lead is extensively used in the preparation of pipes, batteries, and electronic devices [3,4,5]. On the other hand, cadmium is utilized in a range of construction materials, while chromium is used for the production of steel, which is commonly used in kitchen appliances. Mercury is a hazardous metal used in fluorescent lamps and switches, and cadmium is employed in rechargeable batteries [6,7]. The employment of metal ions is a viable approach towards economic development; however, despite its numerous benefits in enhancing the standard of life, it damages the environment [8]. For instance, heavy metals like copper, mercury, cadmium, lead, zinc, and chromium are used in industries for making several materials used in our daily lives. When industry waste is disposed of into the environment, it pollutes the water and air, which further affects human health [9,10]. While metals are crucial for various industries, the negative environmental impacts outweigh their economic benefits. To mitigate their negative impacts, it is essential to control its usage and maintain proper disposal practices. Additionally, exploring alternative materials and technologies that reduce reliance on heavy metals can help promote sustainable economic development. Detecting these toxic metals is essential for curbing their harmful environmental impact. Over the past few decades, CDs have gained considerable attention for their substantial potential in the detection of metal analytes. CDs, with their distinctive chemical, optical, and physical properties, present a promising alternative for metal ion sensing [11]. While traditional synthesis methods for CDs have primarily relied on chemical processes, these approaches often involve harsh chemicals that form complex byproducts and pose additional environmental concerns. Consequently, there is an urgent need to develop straightforward methods for synthesizing cost-effective and biocompatible nanomaterials [12]. Compared with the conventional synthesis techniques of P-CDs, green methods are an economical way to synthesize P-CDs that possess strong luminescence properties and small size and are non-toxic, environmentally friendly, and highly biocompatible. These eco-friendly approaches often utilize natural resources, such as plant extracts and agricultural byproducts, which reduces the cost of toxic chemicals and minimizes energy consumption, significantly reducing reliance on costly and toxic chemicals. By minimizing energy consumption and waste generation, green synthesis not only enhances the environmental profile of carbon dot production but also lowers overall production costs, making it an attractive alternative for large-scale applications. The unique characteristics of P-CDs make it an attractive alternative to broaden their application in several fields. On the other hand, the usage of highly toxic chemicals can be avoided by applying the green chemistry principles for the synthesis of P-CDs. This review aims to provide a straightforward and clear understanding of how plant-derived carbon dots are used to detect heavy metal ions. The study demonstrates the molecular mechanisms by which green synthesized P-CDs interact with various heavy metal ions, focusing on the principles that form the basis for their detection. This review puts stress on the importance of environmentally friendly synthesis methods and the potential of plant-derived P-CDs in achieving efficient detection of toxic metals. To emphasize the importance of our review, we compared it with recent studies in the field of heavy metal detection. Recent comprehensive reviews, including one by Zhang et al. in the year 2024, demonstrated the numerous green synthesis methods for P-CDs, explaining various techniques for the synthesis of P-CDs that have already been explained in various studies. The study of Bo Zhang et al. mainly focused on the development of selective electrochemical detection techniques [13]. In the same year, Khan et al. developed P-CDs and enhanced their efficiency by several modifications for sustainable development. The main focus of this review is on optimizing the different precursors and synthetic mechanisms to explore the distinct characteristics [14]. In the year 2023, Singh et al. investigated the P-CDs as highly sensitive and selective sensors for sensing. The study only focuses on the different mechanisms utilized for sensing [15]. In the same year, Saah et al. analyzed the enhancement of sensing techniques by incorporating foreign materials. The study mainly focuses on composite materials, which are generally an incorporation of organic molecules or metals with P-CDs [16]. Recent comprehensive reviews shed light on many aspects of P-CDs and their applications in heavy metal detection. However, our evaluation stands out by focusing on plant-derived P-CDs, which have gained popularity due to their environmentally friendly techniques and unique features. The current review focuses on the formation of P-CDs using natural precursors and their usefulness in detecting different heavy metal ions present in the environment, as opposed to other reviews that may include a larger range of synthesis methods or applications. Furthermore, we highlight heavy metal ions with the molecular mechanisms underlying metal ion sensing with plant-derived P-CDs, a topic that has received little attention in the existing literature. This specialized focus provides new insights and a full perspective, bridging a gap created by previous reviews.

**Figure 1:**
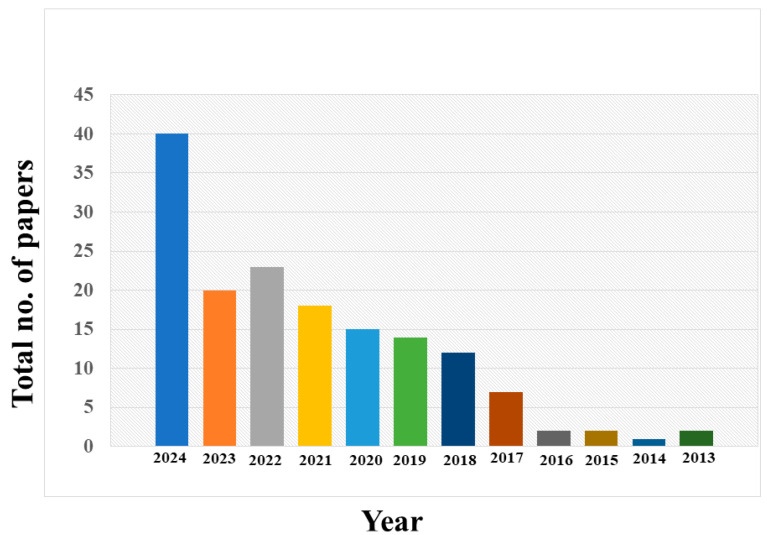
Year-wise research data on detection of heavy metals using green synthesized P-CDs.

To demonstrate the high level of activity and continuous research interest in this area, we included a graph of extracted data from Scopus (Figure 1). The data were extracted from Scopus specifically for the green synthesis of P-CDs, illustrating the number of publications and patents produced annually on heavy metals detection and P-CDs. These data demonstrate the growing interest and rapid advances in this field. Such trends highlight the importance of conducting a thorough and up-to-date review to stay current with the latest advancements and provide a consolidated source of information for researchers and practitioners in the field.

## 2. Synthesis and Structural Properties of P-CDs

Raw materials, including fruits, vegetable peels, seeds, nuts, leaves, and agricultural wastes, are utilized for the preparation of P-CDs. The P-CDs derived from raw materials offer biocompatibility, cost-effectiveness, unique optical properties, and a wide range of applications. Conventionally, P-CDs are prepared using laser ablation and chemical oxidation approaches, which include harsh chemical and toxic precursors. In the modern era, P-P-CDs are prepared using green precursors, which are more sustainable for the environment. The current synthesis techniques include hydrothermal, microwave, and green synthesis methods. The plant extracts contain various phytoconstituents, which act as natural reducing and stabilizing agents. The P-CDs prepared from natural precursors typically consist of spherical-shaped nanostructures that are primarily made of C-atoms, with minor additions from other elements. In these 3D clusters, carbon atoms are mostly grouped in sp^3^-hybridized configurations, with some in sp^2^-hybridized forms [17]. This hybridization provides a large number of surface functional groups to the P-CDs, which frequently have both crystalline and amorphous areas. Although sp^2^ carbon regions contribute to crystalline structure, C-dots often have low total crystallinity. One notable feature of P-P-CDs is their quantum size, which is more pronounced; P-CDs have small particle sizes, typically less than 10 nm [18]. The particle size increases, surface area decreases, and notable red-shift in their fluorescence emission wavelengths is observed. The surface properties of P-CDs are modified by incorporating other materials to enhance the performance. The diverse properties of P-CDs have gained significant interest from researchers. In this review, we utilized natural resources for the formation of P-CDs for the detection of numerous heavy metals present in the environment. P-CDs s have different fluorescence characteristics, which can be greatly affected by a variety of mechanistic processes [19]. These variations are principally caused by interactions among metal ions and the surface functional groups of CQDs, which may diminish or enhance their fluorescence. This contact can initiate the recombination of electron–holes paths, altering the fluorescence properties of P-CDs. Several processes include static quenching, Forster resonance energy transfer (FRET), dynamic quenching, the inner filter effect (IFE), photoinduced electron transfer (PET), and charge transfer. Static quenching occurs when metal ions form non-fluorescent complexes with P-CDs, whereas dynamic quenching involves collisions between excited P-CDs and metal ions, resulting in non-radiative energy loss. FRET involves the transfer of energy from P-CDs to metal ions, which influences emission intensity. The IFE can absorb excitation or emission light, which reduces fluorescence [15]. PET involves the movement of electrons between CQDs and metal ions, which can reduce fluorescence, and charge transfer can change the electronic states of CQDs, affecting fluorescence emission. Understanding these mechanisms is critical for optimizing CQDs for a number of applications, such as imaging, sensing, and environmental monitoring, because they determine how CQDs react to diverse stimuli and situations.

## 3. Application of P-CDs for the Detection of Heavy Metal Ions

P-CDs are gaining the attention of researchers due to their unique fluorescence characteristics that can be easily tailored according to specific sensing applications. P-CDs allowed for sensitive and selective detection. In the subsections, we explore the application of P-CDs for detecting individual metal ions and highlighting their interaction mechanisms.

### 3.1. Iron (Fe^3+^, Fe^2+^)

**Figure 2:**
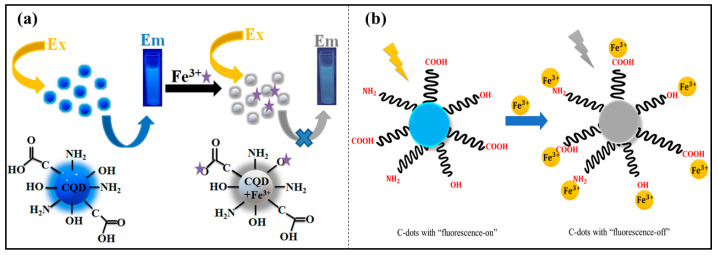
Schematic representation of fluorescence quenching of P-CDs on addition of Fe^3+^ ions (**a**) emission and excitation along with chemical structure, (**b**) Turn-On and Turn-Off mechanism [37].

Iron is one of the most abundant metals iron present in the earth’s crust and poses significant health hazards and environmental concerns when present in high concentration [20,21]. Iron is the most important metal ion for human health, although high levels of iron can be hazardous to the environment, as well as to human beings [22]. It is critical to observe Fe^3+^ levels in the surroundings and biological systems. The high or low concentration of iron causes several disorders and certain kinds of cancers. Iron exists in two possible oxidation states, including Fe^2+^ and Fe^3+^, which are ferrous and ferric, respectively. Out of the two, ferrous is more toxic in nature compared with ferrite. Ferric ions have high reactivity and become easily soluble in water, which allows them to easily enter the water bodies and further contaminate the water sources. Moreover, iron contamination can have a long-term impact on soil quality and crop yields. To mitigate the adverse effects of iron ions, various attempts have been made, of which green synthesis of P-CDs is one of the best alternatives. Since Fe^3+^ detection requires specific, economical, and sensitive technological advances, the introduction of inexpensive and portable fluorescent P-CDs that can detect Fe^3+^ has received lots of interest from researchers. The various green precursors involved in the formation of P-CDs for the detection of iron metal ions are tabulated below. Various studies have been conducted on the detection of iron ions using green precursor-developed P-CDs. For instance, Ge et al. developed N-doped P-CDs using fresh tea leaves for sensing Fe^3+^ ions. The study observed that the surface of N-P-CDs contains hydroxyl, carboxyl, and amino functional groups, which caused quenching by effective chelation/coordination with Fe^3+^ ions. The Fe^3+^ ions exhibit stronger binding affinity with P-CDs as the fluorescence emission intensity decreases with increasing concentration of Fe^3+^ ions. The presence of numerous groups on P-CDs acts as electron donors, and Fe^3+^ ions act as electron acceptors, resulting in the interaction between the Fe^3+^ and P-CDs [23]. Further, Singh et al. investigated P-CDs using the hydrothermal approach for the selective sensing of Fe^2+^ ions. In the study, luminescence quenching occurs as a result of metal ions absorbing the energy of excited electrons. The fluorescence intensity of CQDs depends on the concentration of Fe^2+^ ions, which means that Fe^2+^ acts as a quencher of luminescence in P-CDs. The groups of functional molecules above the surface of P-CDs have a significant binding affinity for Fe^2+^ ions. Amine, hydroxyl, and carboxyl-like groups on the surface of P-CDs contribute to a decrease in fluorescence intensity due to interactions with Fe^2^⁺. The sensing mechanism primarily involves electron–hole recombination involving a non-radiative process and photoluminescence quenching. As electrons are excited to higher energy levels, they interact with the metal ions, leading to non-radiative processes [24]. Further, Diao et al. synthesized P-CDs from the natural precursor Syringa oblata using a hydrothermal approach to sense Fe^3+^ ions. The findings state that fluorescence quenching of the P-CDs occurred due to strong binding interactions between the P-CDs’ functional groups and Fe^3^⁺ ions, with photoelectron transfer leading to reduced fluorescence intensity. The decrease in photoluminescence with increasing Fe^3+^ concentrations indicated high selectivity of the P-CDs for Fe^3+^ ions [25]. Moreover, Nair et al. developed P-CDs from snake gourd peel to detect Fe^3^⁺ ions using household waste as the precursor in a hydrothermal synthesis process. The study demonstrated that hydroxyl, carboxyl, and amino groups on the surface of the P-CDs interact selectively with Fe^3+^ ions, leading to a significant decrease in fluorescence intensity compared with other metal ions, thus highlighting the P-CDs’ specificity for Fe^3+^ [26]. In another investigation, Atchudan et al. synthesized N-doped P-CDs using fruit extract from *Chionanthus retusus* for Fe^3^⁺ ion detection utilizing a hydrothermal technique in the formation of P-CDs. The study observed the quenching mechanism in the presence of Fe^3+^ ions due to the strong binding affinity of nitrogen and oxygen-containing functional groups on the N-P-CDs’ surface. The non-radiative electron transfer process between Fe^3+^ and the N-P-CDs, driven by the strong interaction between hydroxyl and carboxyl groups with Fe^3+^, underscores the high selectivity of the N-P-CDs for Fe^3+^ ions [27]. Kalanidhi et al. developed fluorescent P-CDs from betel leaves, which were nitrogen-doped to enhance the selectivity towards Fe^3^⁺ ions. The study followed a quenching mechanism where strong binding interactions between the P-CDs and functional groups on the surface and Fe^3+^ ions led to coordination interactions. The excited electrons interact with vacant orbitals of Fe^3+^, promoting electron–hole recombination and resulting in fluorescence quenching [28]. In another study, Liu et al. utilized natural precursors like rose-heart radish to synthesize P-CDs via a hydrothermal method for Fe^3+^ sensing. Their findings showed that fluorescence quenching occurred due to strong binding interactions between hydroxyl and phenolic groups on the P-CDs’ surface and Fe^3+^ ions. The coordination interactions facilitated non-radiative electron/hole recombination, with electrons in the excited state transferring to the unfilled orbitals of Fe^3+^, leading to decreased fluorescence intensity with increasing Fe^3^⁺ concentrations [29]. Lastly, Senol et al. developed P-CDs derived from Seville orange using a hydrothermal method for Fe^3^⁺ ion detection. The study reported fluorescence quenching upon the addition of Fe^3^⁺ ions, which exhibited a strong affinity for nitrogen and oxygen atoms on the P-CDs’ surface. The coordination between P-CDs and Fe^3+^ ions was more pronounced compared with other metal ions, and the quenching efficiency, assessed using the Stern–Volmer equation, further confirmed the high sensitivity of the P-CDs towards Fe^3+^ ions [30]. Further, Achadu et al. synthesized P-CDs using expired potato dextrose agar and oxalate for the detection of Fe^3+^. In the study, the authors utilize oxalate on the surface of P-CDs to enhance the fluorescence intensity. The study follows the doping of oxalate on the surface of P-CDs forming ag-oxP-CDs to an electronic and molecular interaction with Fe^3+^ ions as it makes a complex between the ag-oxP-CDs and Fe^3+^. The synergistic interaction between the Fe^3+^ and surface oxalate results in fluorescence enhancement. Also, the study reveals that the interaction results in the inter and intra-induced aggregation that binds the target analytes, resulting in an enhanced fluorescence signal [31]. In another study, Sachdev et al. synthesized P-CDs utilizing the hydrothermal method from coriander leaves for the sensing of Fe^3+^ ions. The as-prepared P-CDs show selectivity towards Fe^3+^ ions due to coordination between the hydroxyl group present on the surface of P-CDs and Fe^3+^ ions. In the study, fluorescence quenching was investigated based on Stern–Volmer graphs. The study reveals that P-CDs are pH-dependent, as the quenching efficiency was found to be low under acidic conditions, resulting in weaker interaction between Fe^3+^ and P-CDs. In the pH range of 7–9, quenching efficiency is high, resulting in stronger interactions between the Fe^3+^ and P-CDs. Thus, the study shows the high selectivity of P-CDs attributed to the detection of Fe^3+^ ions [32]. Yang et al. developed P-CDs from natural precursor honey for the efficient sensing of Fe^3+^ ions. The study follows the binding mechanism in which fluorescence quenching occurs. The authors and his coworkers reveal that the fluorescence quenching of P-CDs occurs due to the binding sites available on the surface because of various functional groups that interact with the Fe^3+^ ions. The coordination interaction between P-CDs and the Fe^3+^ led to the aggregation of particles, which further quenched the P-CDs fluorescence [33]. Sun et al. synthesized carbon dots (P-CDs) from the natural precursor Lycii fructus using a hydrothermal method for Fe^3+^ ion detection. In their study, fluorescence quenching of the P-CDs was attributed to the formation of complexes between the P-CDs’ functional groups and Fe^3+^ ions via coordination interactions. This complex formation enhanced non-radiative electron–hole recombination as electrons transferred from an excited state to the unfilled orbitals of Fe^3^⁺, resulting in fluorescence quenching. The selectivity of P-CDs for the Fe3+ ions was further demonstrated by their incredible interaction affinity with the hydroxyl groups on their surfaces [34]. Similarly, Polatoğlu et al. developed P-CDs from *Kumquat* for Fe^3+^ ion sensing. The study observed a decrease in the fluorescent intensity of P-CDs with increasing Fe^3+^ ion concentrations. It was revealed that the P-CDs exhibited high absorption spectra due to π−π* transitions in the aromatic sp^2^ domains. Gradual fluorescence quenching occurred as the excitation wavelength shifted, further indicating sensitivity to Fe^3+^ ions [35]. Prasasti et al. investigated P-CDs from agricultural waste corncobs via a microwave-assisted method for Fe^3+^ ion detection. The study explored the effect of varying corncob concentrations on metal ion sensing. Results showed that higher corncob concentrations led to a blue shift in the peak wavelength and increased fluorescence intensity. However, a decrease in intensity with increasing Fe^3^⁺ concentrations indicated the P-CDs’ selectivity for Fe^3+^ ions [17]. Likewise, Devi et al. synthesized P-CDs using aloe vera as a natural precursor for Fe^3+^ ion sensing. The P-CDs were characterized by oxygen-containing functional groups such as –COOH and –OH, which contributed to fluorescence quenching. These functional groups exhibited a strong binding affinity towards Fe^3+^ ions. The quenching mechanism was explained using the Stern–Volmer equation, which demonstrated that fluorescence intensity decreased with increasing Fe^3+^ concentrations, confirming the P-CDs’ selectivity for Fe^3+^ ions [36]. Further, Latief et al. demonstrated P-CDs from gelatin as a green material for Fe^3+^ ion detection. The study found that fluorescence quenching of the P-CDs occurred upon the addition of Fe^3+^ owing to electron transfer between the P-CDs’ surface functional groups and Fe^3+^ ions. Oxygen-containing groups, particularly –OH and –COOH, demonstrated strong binding affinity to Fe^3^⁺, leading to coordination interactions that facilitated electron transfer between the unfilled orbitals of Fe^3+^ and the P-CDs, resulting in fluorescence quenching. Compared with the other metal ions like Ni^2+^ and Cu^2+^, Fe^3+^ ions have stronger affinity because other metal ions have weaker electrophilic ability, and they bind to the electron-donating groups [37]. The schematic representation of the detection of Fe^3+^ ions is shown in Figure 2. Furthermore, Ran et al. synthesized P-CDs using natural precursor chloroplast via hydrothermal method for the effective detection of Fe^3+^ ions. The study depicts the cost-effective, eco-friendly approach for Fe^3+^ ions, which follows the static quenching mechanism in which electron transfer takes place [38]. In 2023, George et al. synthesized P-CDs derived from papaya seeds for the efficient detection of Fe^3+^ ions in aqueous solution. The study shows that there is a complex formation, and the energy transfer takes place between the d-orbital of Fe^3+^ metal ions and functional groups present on the surface of P-CDs. Due to the formation of the complex, the electrons are excited from the lower energy state of P-CDs to the d-orbital of metal ions, which further shows the quenching phenomenon. The quenching behavior of Fe^3+^ ions indicates the strong absorption affinity of Fe^3+^ ions towards P-CDs [39]. In Table 1, we present a summary of various natural precursors used for the synthesis of carbon dots (P-CDs), including their particle sizes and their impact on the detection limits for Fe^2^⁺ and Fe^3^⁺ ions. Table 1 below shows how various precursors affect the size of the P-CDs and their sensitivity in detecting iron ions.

Xia et al. demonstrated waste-derived P-CDs using the hydrothermal technique for Fe^3+^ and Cr^6+^ ions detection. The study reveals that the ground state complex formed between the Fe^3+^ and oxygen-containing functional groups of P-CDs facilitates fluorescence inhibition. The quenching occurs due to interaction between the hydrophilic carboxyl, hydroxyl, and amine groups and Fe^3+^ ions. The stronger binding affinity of Fe^3+^ towards the P-CDs shows the selective recognition of Fe^3+^ ions [42].

### 3.2. Mercury (Hg^2+^)

**Figure 3:**
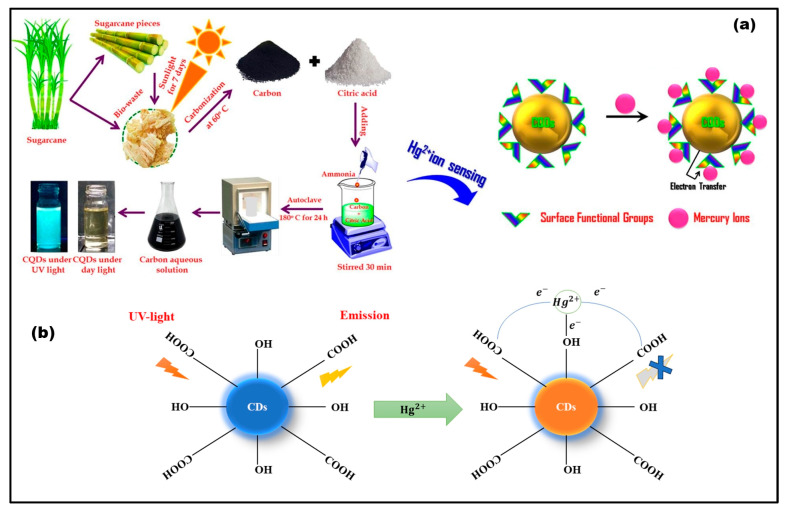
(**a**) Schematic representation of sugarcane derived CDs for the detection of Hg^2+^ ions [51]. (**b**) Illustration of inhibition of fluorescence spectra of P-CDs on addition of Hg^2+^.

Mercury is a prominent heavy metal with catastrophic consequences on both the surroundings and human health [45,46]. It comes in a variety of forms and can cause significant harm when released into the environment. Mercury in our environment exists in three oxidation states: elemental (Hg^0^), mercuric (Hg^2+^), and mercurous (Hg^+^). Mercuric mercury (Hg^2+^) is generally considered to be the most hazardous for the environment and human health. It can easily interact with biological systems and impair the functioning of cells, particularly in the neurological system. Mercury exposure is known to cause severe neurological damage, renal dysfunction, respiratory complications, and, in extreme cases, death. According to WHO, the permissible concentration of mercury in drinking water is 1 mg/L [47]. Given trace levels of metal ions like mercury in the environment, there has been significant research and development of materials for their detection. Among these, green synthesized P-CDs have proven particularly effective owing to their ability to form complex structures with metal ions. In a study conducted by Yu et al., a fluorescent, water-soluble carbon dot-based sensor was synthesized using a green, cost-effective hydrothermal approach, with Jinhua bergamot serving as the natural carbon precursor. These carbon dots (P-CDs) demonstrated the capability to detect Hg^2+^ and Fe^2+^ ions, attributed to the presence of amino and carboxyl functional groups on their surface. These groups exhibited strong binding affinity and rapid chelation kinetics with heavy metal ions like Hg^2+^ and Fe^3+^, enhancing the sensor’s selectivity [12]. Similarly, Lu et al. synthesized P-CDs from pomelo peels using a hydrothermal method specifically for Hg^2+^ ion detection. The quenching mechanism was investigated using the Stern–Volmer equation, which confirmed that Hg^2+^ ions could efficiently quench the fluorescence of P-CDs due to their high binding affinity to the carboxyl groups present on the P-CDs’ surfaces compared with other metal ions [48]. Further research by Gaddam et al. utilized camphor as a carbon source to produce P-CDs for Hg^2+^ detection in water samples. A marked reduction in fluorescence intensity was observed upon Hg^2+^ addition, with the quenching explained through non-radiative electron–hole recombination. The Stern–Volmer analysis further supported the direct relationship between Hg^2^⁺ concentration and the quenching effect [49]. In another study, Huang et al. developed nitrogen-doped carbon dots (N-P-CDs) using strawberry juice as a precursor through a hydrothermal synthesis method, which demonstrated high sensitivity and selectivity toward Hg^2+^ detection [50]. Moreover, Kasinathan et al. synthesized carbon quantum dots (CQDs) from sugarcane waste using a hydrothermal process, showing that Hg^2^⁺ ions caused significant quenching of the CQDs’ fluorescence (Figure 3). This effect was attributed to electrostatic interactions between the functional groups on the CQD surface, such as hydroxyl and amine groups, and Hg^2+^ ions, leading to excellent selectivity and sensitivity for Hg^2^⁺ detection [51]. In a similar manner, Qin et al. employed a microwave-assisted method to produce P-CDs from flour for Hg^2+^ detection, with the quenching effect being linked to non-radiative electron transfer between Hg^2^⁺ ions and the P-CDs. The study highlighted the pH-dependency of the interaction, where the binding affinity between Hg^2+^ and P-CDs increased at higher pH, enhancing the stability of the formed complex [52]. Furthermore, Xie et al. synthesized nitrogen-doped P-CDs using peas as a carbon source and ethanediamine as a nitrogen source via hydrothermal synthesis. The introduction of nitrogenous auxochromes, including C-NH_2_, OC-NH_2_, and C–N–C, improved fluorescence intensity. However, the addition of Hg^2^⁺ ions resulted in fluorescence quenching, attributed to non-radiative electron transfer between the vacant d-orbitals of Hg^2+^ and the N-P-CDs, as supported by complex formation between N-P-CDs and Hg^2+^ (Figure 3). The non-radiative electron transfer occurs between the vacant d-orbital of Hg^2+^ and N-P-CDs, resulting in the quenching of N-P-CDs fluorescence intensity [53]. Similarly, Askari et al. investigated the “turn-on” fluorescence P-CDs for the efficient detection of Hg^2+^ ions [54]. In another study, Li et al. developed P-CDs using bovine serum albumin for the efficient detection of Hg^2+^ ion. The study shows the appearance of no new peak after the addition of Hg^2+^ in silicon-doped P-CDs, which indicates the static quenching effect. The fluorescence quenching of Si-P-CDs was observed upon the addition of Hg^2^⁺, resulting from the internal filtering effect associated with the excitation spectrum. The transmission electron spectroscopy (TEM) indicates that the aggregation of Si-P-CDs leads to fluorescence quenching [55]. In Table 2, we provide a summary of various natural precursors used for the synthesis of P-CDs, highlighting their particle sizes and their influence on detection limits for Hg^2+^ ions. This table illustrates how different precursors affect both the size of the P-CDs and their sensitivity in detecting iron ions.

### 3.3. Copper (Cu^2+^, Cu^+^)

Copper is an essential trace element that is vastly employed for the normal functioning of several biological processes in humans and animals as well [56,57]. However, excessive exposure to these harmful ions can cause disorders, including kidney failure, Parkinson’s disease, liver damage, and Alzheimer’s [58]. It is crucial to manage its usage for the safety of the ecosystem and human health. Copper exists in two oxidation states, +1 and +2, of which Cu^2+^ is the toxic and highly stable metal ion present in our surroundings. Although copper toxicity is very low because the human body regulates copper levels, its exposure to high concentrations causes several hazards, including nausea, kidney damage, vomiting, diarrhea, and gastrointestinal diseases. To manage copper levels, it is essential to detect it and mitigate its presence in the environment. In line with this, Tan et al. developed P-CDs using a thermal pyrolysis method with sago waste as a natural precursor for the detection of Cu^2+^ and Pb^2+^ ions. The study revealed that the fluorescence intensity of the P-CDs was significantly quenched in the presence of Cu^2+^ and Pb^2+^ compared with other metal ions. The researchers also evaluated the fluorescence quenching effects of other metal ions such as Hg^2+^, Zn^2+^, and Ca^2+^; however, due to the diamagnetic properties of these ions, the quenching effect was notably less pronounced; it eliminates the quenching mechanism with P-CDs. The Pb^2+^ and Cu^2+^ showed the highest absorption affinity with C-dots due to the non-radiative pathway [59]. Liu et al. investigated P-CDs using bamboo waste leaves for Cu^2+^ recognition ions. The study analyses the inhibition mechanism under controlled pH conditions. The quenching is observed under the pH 4–6. Thus, only at pH 4 is the highest fluorescence emission observed, and the Cu^2+^ ion results in significant inhibition of BPEI-CQDs compared with other metal ions that show negligible responses, which is due to the chelation of both the sensors and toxic elements [60]. Further, Sanni et al. developed fluorescent P-CDs for Cu^2+^ recognition via microwave-assisted pyrolysis. In the study, pinecone is employed in P-CD synthesis. The study demonstrates a significant interaction between the carboxyl, hydroxyl, and amino groups on P-CDs’ surface. This leads to the formation of a stable complex that facilitates effective electron transfer and enhances non-radiative electron–hole recombination. As a result, there is a pronounced fluorescence quenching of the P-CDs [61]. Gedda et al. synthesized environmentally friendly carbon dots for the selective detection of Cu^2+^ ions using a green synthesis approach. In the study, prawn shells were employed as the natural precursor for the production of P-CDs, which demonstrated effective sensing of Cu^2+^ ions. The quenching mechanism was attributed to the inner filter effect, where Cu^2+^ ions interact with amine groups on the P-CDs, forming a cupric amine complex. To confirm this mechanism, the authors compared the fluorescence of CD solutions with and without Cu^2+^ ions. The observed reduction in fluorescence intensity confirmed the role of the cupric amine complex in the selective sensing of Cu^2+^ ions [62]. To highlight the significance of different precursors, Table 3 below provides a clear context for the sensing of Cu^2+^ ions using P-CDs. This table illustrates the significant variations in detection efficiency based on selected precursors.

### 3.4. Chromium

Chromium is a transition metal with an assortment of industrial applications, including manufacturing, colorants, and alloys. However, the element’s presence in the environment, especially when it occurs in the form of chromium ions, can have serious consequences for the environment and the wellness of humans. Chromium exists in numerous oxidation states, the most frequent and environmentally relevant of which are hexavalent chromium Cr^6+^ and trivalent chromium Cr^3+^. However, hexavalent chromium Cr^6+^ has been identified for its potentially hazardous and carcinogenic aspects, resulting in an important concern in a variety of industries [65,66]. Chromium is highly hazardous and still used in various industries, including industrial, domestic, agricultural, medicinal, electronics, and optical coatings. Chromium plays a significant role in the manufacturing of kitchenware, as industries heavily utilize it in stainless steel to enhance corrosion resistance and durability. Various knives and cutlery items use chromium to increase sharpness and prevent rust [67,68]. The hexavalent chromium Cr^6+^ oxidation state is considered more toxic, as it is highly reactive and undergoes redox reactions with body cells, causing carcinogenic diseases. The International Agency for Research on Cancer (IARC) declares the Cr^6+^ ions as carcinogenic compounds [69]. Moreover, it is highly soluble and mobile in an aqueous environment, leaching into the river water and contaminating the aqueous environment. Many attempts have been made to detect even a trace amount of Cr^6+^. In another study, Liu et al. synthesized P-CDs using the hydrothermal method from chocolate as a carbon source for the detection of Pb^2+^ ions. In this work, it was observed that the fluorescence intensity of P-CDs is decreased after the addition of Pb^2+^ ions into the P-CDs solution. The chelation occurs between the oxygen-containing groups present on the surface of P-CDs and Pb^2+^ ions, which enhances the non-radiative recombination of charge transfer of electrons. Also, the study confirms that the fluorescence intensity of P-CDs is inversely proportional to the concentration of Pb^2+^ by the Stern–Volmer equation. The selectivity of P-CDs towards Pb^2+^ ions was also observed using other metal ions, but the quenching effect was negligible with other metal ions [70]. Further, Kumar et al. investigated the synthesis of P-CDs from tulsi leaves as a carbon source. The authors and their coworkers utilize a one-step hydrothermal method for the synthesis of highly specific and selective detection of Pb^2+^ ions. The study highlights that the significant selectivity is due to a high affinity for binding among the empty d-orbital of Pb^2+^ ions and the functional group amine present on the P-CDs surface. In comparison with other metal ions, the nitrogen atom of the amine group gives an electron pair to the unoccupied d-orbital of lead ions via nonradiative electron transfer, which is simple and allows for surface complexation [71]. Moving forward, Boobalan et al. synthesized P-CDs from natural source mushrooms via the hydrothermal technique for the selective sensing of Pb^2+^ ions. The carbon dot contains amino acids and polysaccharides on the surface, because the carbon source mushroom is rich in a variety of nutrients that contain essential amino acids and polysaccharides. When the addition of Pb^2+^ ions takes place in the C-dot, the complex formed with the hydroxyl and carboxyl group, which formed a complex like lead hydroxide and carboxylate, quenched the C-dots. The various metal ions added in the solution of P-CDs to check the selectivity reveal that C-dots show selective binding towards the Pb^2+^ ions, forming the strong coordination bond between the functional group P-CDs and Pb^2+^ ions [72]. Further, Wee et al. synthesized P-CDs using the acid hydrolysis method from bovine serum albumin for the detection of Pb^2+^ ions. The study follows the fluorescence quenching mechanism in which non-radiative recombination of electron transfer takes place from the valance band to the conduction band. The study reveals that there is no shift observed on the emission peak, resulting in the mechanism attributed to electron transfer [73]. In similar work, Xu et al. developed P-CDs using a hydrothermal method derived from ginkgo biloba leaf extract as a carbon source for the sensing of Pb^2+^ ion. In the study, the detection efficacy is enhanced by introducing the flavonoid-like moieties into P-CDs. The high sensitivity and selectivity of P-CDs towards Pb^2+^ metal ions is due to the doping of an agarose hydrogel. The results were confirmed using river water samples by targeting the metal ion [74].

**Figure 4:**
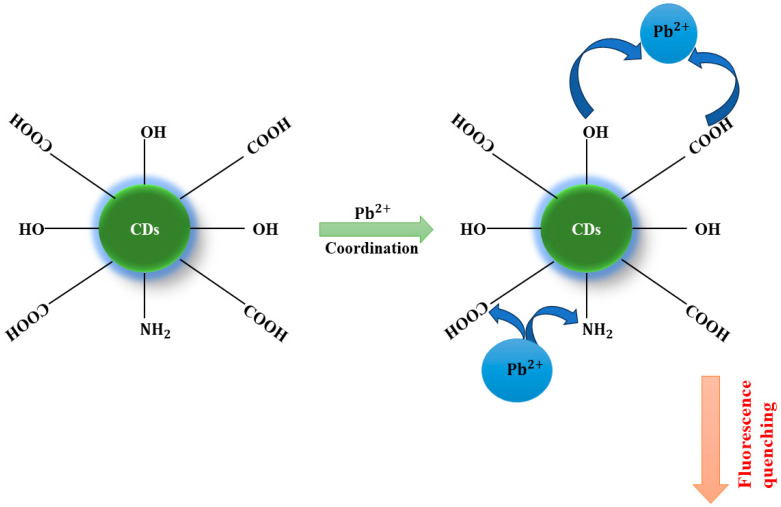
Schematic representation of fluorescence quenching of P-CDs by detection of Pb^2+^ ion.

For instance, Bandi et al. developed a fluorescent-N-doped P-CDs sensor for sensing Pb^2+^ ions. The author and his coworkers utilize a simple one-step green synthesis method using Lantana camara berries as a natural precursor for synthesizing fluorescent P-CDs. The synthesized N-P-CDs exhibit strong fluorescence that is stable in various optimal conditions; also, it is highly selective and sensitive to the effective detection of Pb^2+^. In the study, kinetic experiments indicate that interaction between the Pb^2+^ and functional group present on NP-CDs is very fast as the addition of Pb^2+^ ions in P-CDs shows a stable decrease in emission intensity of NP-CDs. The study investigates the mechanism followed by fluorescence quenching, and the dynamic and static quenching using Stern–Volmer (SV) plots at different conditions reveals the varying quenching according to the variable temperature. The high selectivity obtained is due to the binding affinity of the surface functional groups with Pb^2+^, which facilitates the non-radiative electron transfer from the excited state of NP-CDs to the vacant d-orbital of Pb^2+^ and results in quenching of fluorescence. The schematic representation of fluorescence quenching is demonstrated in Figure 4. Likewise, real water and biological sample analyses have been conducted [75]. The comparative analysis of the detection of Pb^2+^ ion is presented in Table 4, listed below, mentioning the different parameters of various studies.

### 3.5. Co(II)

**Figure 5:**
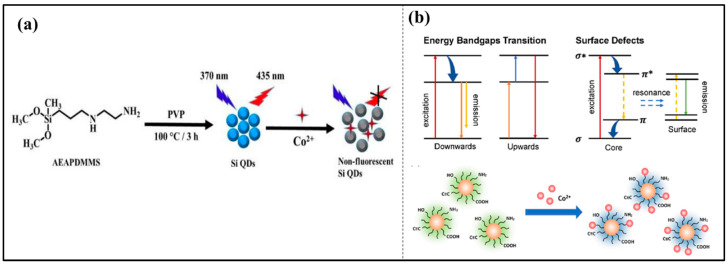
(**a**) Schematic representation of fluorescent Si-P-CDs for the detection of Co^2+^ ions [80]. (**b**) Mechanistic representation of photoluminescence of carbon dots (P-CDs) illustrating HOMO-LUMO transitions and fluorescence sensing of Co^2^⁺ [81].

Cobalt is a well-known transition metal used in various industries for making high-strength alloys and batteries [78]. Cobalt is present in the environment in several different oxidation states, including Co^2+^ and Co^3+^. Co^2+^ is the most stable oxidation state, resistant in water bodies interacting with biological systems. It is an essential element for human health, but its intake at high concentrations poses several respiratory diseases, neurological disorders, and cardiovascular diseases. Various sensing techniques, including chromatography, electrochemistry, and spectroscopy, have been introduced to detect the presence of Co^2+^. Since all these techniques are time-consuming and expensive, green synthesized P-CDs are one of the best alternatives to detect heavy metal ions, as they are inexpensive and selective towards particular metal ions. For instance, Hu et al. synthesized P-CDs derived from flax straw as a carbon source using hydrothermal methods for the detection of Co^2+^ and Cr^6+^ ions. The study follows the inner filter effect-induced fluorescence quenching, which is due to static quenching. The decrease in fluorescence intensity of P-CDs after the addition of metal ions shows the formation of the complex between the functional groups present on P-CDs and metal ions. The Co^2+^ and Cr^6+^ show stronger binding affinity towards the functional group of P-CDs, resulting in fluorescence quenching. The high concentration of Co^2+^ in P-CDs chelates and yields the inner filter effect, which further results in the quenching of fluorescence [79]. In another study, Sullam et al. synthesized P-CDs doped with silicon for the detection of Co^2+^ ions using the hydrothermal method. As per studies, there must be no overlap between the P-CDs and quencher for the occurrence of the inner filter effect and fluorescence energy transfer. The study reveals that the static quenching effect plays a vital role in the fluorescence quenching to form a stable compound [80]. The schematic representation of the detection of Co^2+^ ions is shown in Figure 5. In another study, Wu et al. investigated amine-doped P-CDs for the efficient detection of Co^2^⁺ ions. The mechanism of fluorescence emission was explained through the FRET effect, where light excitation causes emissions from both energy bandgap transitions. The presence of carboxyl groups maintains conjugation, which primarily contributes to the increase in photoluminescence. The sensing of Co^2^⁺ occurs via quenching due to the binding between the surface amine groups, involving the inner filter effect (IFE). The mechanism of study involving energy bandgap transitions, surface defects, and sensing of Co^2+^ ions is illustrated in Figure 5b [81]. Zhao et al. derived C-dot from natural precursor kelp for the detection of Cobalt (II). The authors utilize a microwave-assisted method for the formation of CDs using ethylenediamine as a nitrogen dopant. The synthesized CDs exhibit good water solubility and sensitivity toward the detection of Co^2+^ ions. The research demonstrates that the quenching takes place in CDs with the addition of Co^2+^. The quenching process complied with the inner filter effect since there was evident overlapping of the UV absorption spectra of Co^2+^ and the emission spectrum of CDs [82]. In this regard Table 5, listed below, presents the difference in various parameters in preparation of CDs using natural precursors for the detection of Co^2+^ ions.

### 3.6. Cd(II)

**Figure 6:**
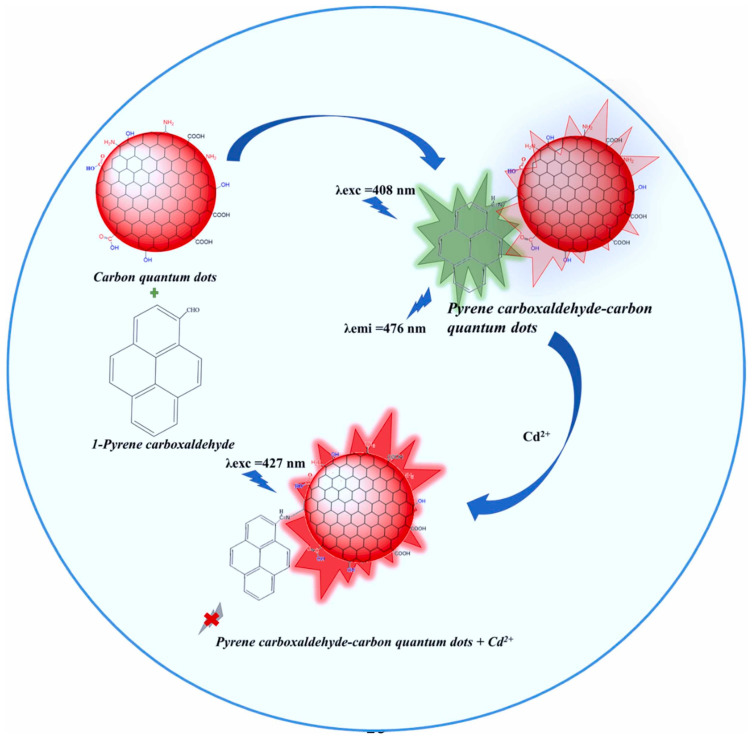
Schematic representation of the mechanism of selective sensing of Cd^2+^ ions using water amaranth leaves derived CDs [87].

Cadmium is a very toxic heavy metal that poses tremendous hazards to both individuals and the environment [85]. It is widely utilized in an array of industrial applications, including battery manufacturing, electroplating, the textile sector, and plastic production. A large amount of industrial discharge is released into the rivers, which pollutes the air and water. In recent years, numerous phosphate fertilizers have been used in agriculture, which causes cadmium to enter the soil and contaminate crops. Cadmium exists in several different oxidation states, of which Cd^2+^ is the most common one, as it is highly water-soluble and readily interacts with biological systems. Cadmium is one of the most toxic metal ions, causing various kinds of health problems, including respiratory issues, kidney damage, and reproductive disorders. The exposure to cadmium causes several risks to human health and the environment. Various analytical processes, including ICP-AES, ICP-MS, and other spectrometric techniques, have been utilized, but these techniques have high costs due to expensive instruments. To address these kinds of issues, fluorescence sensors are developed using natural precursors-derived P-CDs. For instance, Pandey et al. investigated the green P-CDs from Murraya koenigii leaves for the detection of Cd^2+^ ions. In the study, the fluorescence of P-CDs is quenched due to ligand–metal charge transfer. The P-CDs surface contains amino and carboxyl groups that bind with Cd^2+^ ions. P-CDs can contribute electron pairs from the excited state to the empty d-orbital of the Cd^2+^ ion, further quenching the fluorescence intensity of P-CDs. The study reveals that dynamic quenching occurred due to a decrease in the average lifetime of P-CDs. The decrease in absorption intensity confirms that dynamic quenching followed. The high binding affinity between the Cd^2+^ ions and functional groups present on P-CDs indicates the selectivity of P-CDs towards Cd^2+^ ions [86]. In another study, Keerthana et al. investigated the P-CDs derived from amaranth leaves as a ratiometric sensor for the efficient detection of Cd^2^⁺. The study found that after adding Cd^2^⁺, a decrease in fluorescence was observed, indicating that the study follows the inner filter effect. The predominant FRET mechanism occurred between the electron-rich nitrogen group in pyrene carboxaldehyde-carbon quantum dots (PC-P-CDs) and the electron-deficient group of Cd^2^⁺ metal ions (Figure 6) [87]. In another study, Yan et al. developed a fluorescent probe “on–off” sensor for the detection of Cd^2+^ ions. The study follows the internal charge transfer, due to which emission spectra improve and the transfer of electrons occurs [88].

Table 6 provides a comparison of results using various precursors. It details the detection limits, sizes of carbon dots (P-CDs), concentration ranges, and quantum yields.

### 3.7. Ag(I)

Silver has three oxidation states: Ag(I), Ag(II), and Ag(III). Among all three, Ag(I) is the most stable oxidation state of silver and is highly soluble in water, functioning directly with biological processes. According to the World Health Organization, Ag is a worldwide pollutant that causes a variety of problems to human health and the aquatic environment [58]. Silver ions inhibit enzyme processes, resulting in cytotoxicity and genotoxicity, which can damage cellular function. Silver contamination in the environment occurs from a variety of causes, including wastewater discharge and extraction activities. To address these issues, various sensing techniques, including Surface-enhanced Raman spectroscopy (SERS), ICP-AES, and ICP-MS, have been utilized, but the instrumentation is expensive and does not produce accurate results. To overcome these limitations, P-CDs have been utilized and synthesized using green precursor, and it is effective and selective to detect the Ag^+^ ions. For instance, Arumugam et al. synthesized P-CDs from broccoli as natural precursors using hydrothermal methods for the detection of Ag^+^ ions. The surface of P-CDs is occupied with functional groups like carboxyl, hydroxyl, and amine, which interact with the Ag^+^ ion. The study reveals that the energy transfer takes place between the oxygen-containing functional groups on the surface of P-CDs and Ag^+^, which forms a complex resulting in the luminescence quenching of P-CDs [90]. Similarly, Akhgari et al. developed nitrogen-doped P-CDs using pomegranate juice and ammonium hydroxide as natural precursors via hydrothermal method. The study follows the mechanism based on the inner filter effect in which fluorescence quenching of P-CDs takes place. Electrostatic attraction occurs among the functional groups present on the surface of P-CDs and Ag^+^ ions. The fluorescence intensity of P-CDs is sensitive to Ag^+^ ions as the increase in concentration of Ag^+^ ions shows the decrease in intensity of P-CDs shown by the Stern–Volmer equation [91]. Further, Suryawanshi et al. synthesized P-CDs using hydrothermal and pyrolysis methods from the waste biomass as a natural precursor for the detection of Ag^+^ ions. The study shows the selectivity towards Ag^+^ ions by an on–off–on mechanism. The surface of graphene quantum dots (GQDs) is occupied with carboxyl and epoxy groups, which follow the non-radiative recombination of electron transfer. However, after the functionalization of P-CDs with an amine group, the groups present on the surface get replaced by CONH_2_ and CNH_2_, which changes the path and shows fluorescence quenching only for Ag^+^ ions [92].

Li et al. developed P-CDs from guanine natural precursors via a microwave-assisted approach for the sensing of Ag^+^ ions. In the study, the G-CD surface exhibits multiple recognition sites for the detection of metal ions. The guanosine 50-monophosphate group present on the surface provides binding sites to Ag^+^ due to strong coordination interactions between them. To confirm whether guanine provides active sites for binding, the authors synthesized nucleotide-derived P-CDs, which have high coordination ability with groups like phosphate and base. The study calculates the density functional theory to check the binding ability of the base with Ag^+^. The data indicate that G-P-CDs have strong binding affinity because of the non-radiative electron migration from the excited to empty orbitals of Ag^+^ [93]. A large number of case studies were analyzed, and Table 7 presents the differences in various parameters using natural precursors for the detection of Ag⁺ ions.

### 3.8. Zn^2+^, Al^3+^, Au^3+^, V^5+^, and Ru^3+^

Zinc is an essential trace element for biological systems, but its high presence makes it toxic [94]. Due to several industrial activities, including smelting, mining, and metal plating, zinc is released in large amounts into the environment. Chronic exposure to zinc causes neurological problems and respiratory issues, and high intake causes diarrhea, vomiting, and nausea. To mitigate its adverse effects, various studies have been carried out to detect its presence in the environment. For instance, Jayan et al. developed P-CDs using coconut water as a natural precursor for the detection of Zn^2+^. In the study, the authors follow the “on–off” mechanism for the detection of Zn^2+^ ions. This strategy works well to enhance the selectivity of P-CDs towards Zn^2+^ ions. The fluorescence of P-CDs is active at first, or we can say fluorescence is “on”. After the addition of Zn^2+^ ions in the P-CDs solution, the quenching of the fluorescence of P-CDs occurs. The decrease in photoluminescence intensity and quenching of fluorescence of P-CDs after the addition of Zn^2+^ ions indicate the selectivity of P-CDs towards Zn^2+^ ions [95]. Aluminum is among the most abundant elements in the Earth’s crust and exists in the environment in three oxidation states (Al^0^, Al^+3^, Al^+1^). The most stable oxidation state of aluminum is Al^3+^, which affects the environment and human health. Al^3+^ causes health-hazardous diseases like respiratory issues, Breast cancer, and bone softening. The safe intake limit of Al^3+^ is 3–10 mg per day based on body weight. The use of aluminum in several industrial activities causes environmental damage and affects human health. To control the release of harmful metal ions, numerous studies have been conducted to identify their existence. For instance, Bhamore et al. developed carbon dots derived from the natural precursor Pyrus pyrifolia (pear) fruit utilizing the hydrothermal method for the detection of Al^3+^ ions. The enhanced sensing of fluorescent P-CDs is due to the various functional groups present in P-CDs. The functional group present in P-CDs forms a complex with Al^3+^ ions, which makes P-CDs selective for the detection of Al^3+^. The studies reveal that compared with other heavy metal ions, the intensity of P-CDs increases with the addition of Al^3+^ ions, showing significant selectivity and sensitivity toward detection [96]. Moreover, Arumugham et al. synthesized P-CDs from *Catharanthus roseus* leaves using a hydrothermal method for the detection of Al^3+^ and Fe^3+^ ions. The study observed that the fluorescence mechanism, governed by chelation, could either be enhanced or quenched depending on the metal ion interaction. Kinetic analysis indicated that the reduced fluorescence lifetime in the CD-Fe^3+^ complex was due to electron transfer from the excited P-CDs to the vacant orbitals of Fe^3+^. This process led to non-radiative electron–hole recombination, resulting in aggregation-induced emission quenching in the CD-Fe^3+^ system. In contrast to Al^3+^, fluorescence duration increases due to electrostatic interaction, and coordination of Al^3+^ ions with CQD produces CQD-Al^3+^ aggregates from conjugated amorphous masses, which leads to aggregation-induced high FL emission. This shows P-CDs’ effective selectivity for Fe^3+^ and Al^3+^ ions [97]. Further, Zou et al. synthesized highly fluorescent C-dots for the sensing of Al^3+^ using hydrothermal methods involving citric acid and diethylenetriamine. In the study, a ratiometric fluorescence sensor is developed in the process, where P-CDs act as donors and Al^3+^ acts as acceptors based on fluorescence energy transfer. In the study, the sensitive and selective binding towards Al^3+^ is due to effective coordination and a high extent of overlapping between the acceptor and donor atoms. The presence of gold (Au^3+^) exhibits more toxicity to human health compared with metallic gold due to its high reactivity and the capability of gold to produce reactive oxidation species, which damage the cellular redox balance. To detect the presence of Au^3+^ in the environment, green synthesized P-CDs have been utilized [98]. For instance, Liao et al. developed N-doped P-CDs using natural precursor peach gum for the effective and efficient detection of Au^3+^ ions utilizing the hydrothermal carbonization method. The study follows the synergetic interaction between the Au^3+^ and N-P-CDs and FRET, which cause electron transfer to each other after the addition of Au^3+^ ions. The resulting fluorescence quenching is due to the FRET quenching effect. The results reveal that the enhancement in the absorption band results in the formation of Au particles, which work as acceptors to absorb the fluorescence of N-P-CDs. Moreover, multi-ion detection has been performed using a quenching mechanism [99]. Mohandoss et al. investigated P-CDs produced from naturally available source Hibiscus tea waste for the detection of Ag^+^, Cd^2+^, and Cr^3+^ ions using a hydrothermal approach. In the study, the highly selective and sensitive detection takes place due to nitrogen and boron-doped P-CDs. The fluorescence enhancement of NB-P-CDs after the addition of metal ions like Ag^+^, Cd^2+^, and Cr^3+^ is due to the stronger binding affinity of metal ions towards the surface functionalized P-CDs. The study reveals that the presence of nitrogen and oxygen groups on the surface of P-CDs provides active binding sites for the heavy metal ions, which results in the enhancement in detection [100]. Further, Hoan et al. demonstrated green-developed P-CDs from lemon for the detection of V^5+^ ions. In the study, the authors observed a decrease in the luminescence intensity of Fe^2+^, Fe^3+,^ and V^5+^ ions. The presence of these ions affects the luminescence intensity of P-CDs acting as chelators. Out of the three, V^5+^ shows a remarkable decrease in intensity compared with other metal ions. The coordination interaction between the presence of functional hydroxyl and carboxyl groups on the surface of P-CDs and V^5+^ ions changes the electronic structure of P-CDs. The non-radiative recombination of charge transfer takes place. The stronger binding affinity and stability of V^5+^ ions towards P-CDs shows the selectivity towards P-CDs [101]. Moreover, Hashemi et al. investigated green P-CDs from the red beetroot for the detection of Pd^2+^ ions. The study shows that quenching of fluorescence occurs due to coordination interaction between the functional groups present on the surface of P-CDs and Pd^2+^ ions. In the study, the mechanism of static quenching is followed in which chelation occurs due to the stronger binding affinity of Pd^2+^ ions with P-CDs and the formation of a complex in the ground state, which is due to the interaction between groups on P-CDs and Pd^2+^ [102]. Likewise, Menglin Chen et al. investigated P-CDs for the selective detection of Ru^3+^ ions by hydrothermal approach. The study follows the two mechanisms of the photoluminescence phenomenon, including (i) the transition between the band gap based on conjugate structure and (ii) the presence of surface defects in P-CDs. The surface of P-CDs is occupied by functional groups that coordinate with Ru^3+^ ions [103].

Furthermore, other precious metals such as platinum (Pt), iridium (Ir), osmium (Os), rhodium (Rh), and rhenium (Re) are considered less toxic, with minimal human exposure. The adverse impacts of these metals are not well documented. However, some forms of these metals and their complexes can be toxic, such as cisplatin, a toxic form of platinum (II) complexes, and rhodium chloride, among others. A few studies have been conducted on the detection of these metals and their complexes [104,105].

Unfortunately, no studies have been found on P-CDs for the detection of these metal ions and their complexes. Therefore, future research should focus on the detection of these metals and their complexes using P-CDs.

The different metal ion detections using P-CDs are compiled in Table 8, along with their synthesis method, detection limit, size, and other experimental parameters.

## 4. Industrial Applications of Plant-Derived Carbon Dots for Heavy Metal Ion Detection

The increasing interest in plant-derived carbon dots (P-CDs) stems from academic research into several kinds of industrial applications. Green synthesized P-CDs possess distinctive characteristics, including biocompatibility, strong luminescence, and sustainability, which renders them ideal for tackling the problem of the contamination of heavy metals in an array of domains. The industrial applications of green-derived P-CDs are impactful and broad because P-CDs offer a non-toxic and cost-effective alternative approach to real-time analysis. P-CDs can be incorporated into contemporary filtering systems for wastewater treatment and sludge analysis, assisting industries in managing heavy metal discharges while adhering to environmental requirements. Furthermore, in industries like battery manufacture and metal plating, P-CDs can be used in wearable sensors to monitor worker exposure to heavy metals and ensure occupational safety, as well as in product quality control to ensure that consumer goods satisfy safety standards. P-CDs also show potential for enhancing food safety by detecting metal contamination in food products, as well as promising better agricultural techniques through assessing herbicides and fertilizers. CD-based assessment kits can also help authorities execute the on-site verification of compliance more efficiently. Overall, the use of plant-derived P-CDs in various industrial contexts not only improves detection capabilities but also corresponds with sustainable practices by lowering reliance on hazardous chemicals and aiding regulatory compliance, contributing to a safer and more sustainable future.

## 5. Conclusions and Future Perspectives

This review highlights the study of the green synthesis of P-CDs using plant-based natural precursors that are non-toxic and have various physical and chemical properties used for the sensing of contaminants, like several heavy metal ions from the environment. Nowadays, P-CDs are fascinating materials, acting as sensing probes for various heavy metal ions. The green method to synthesize P-CDs is less toxic, environmentally friendly, cost-effective, and has excellent photoluminescence properties, which is useful for the selective detection of metal analytes. This study shows the various natural precursors used to synthesize P-CDs vary with different properties and functional groups present on their surface. The properties can be easily customized according to the selective detection of particular metal ions. The stability and functionality of P-CDs also vary according to the different approaches utilized for their fabrication. Researchers are still searching for the exact mechanism of synthesis of P-CDs with varying functional groups with the usage of different natural resources. Nevertheless, the quenching mechanism of the detection of metal ions is not yet clear. The green synthesis method has various advantages, but there is still an urgent need to improve the quantum yield of P-CDs, which is very low compared with the chemical synthesis methods. P-CDs show different photo-luminescence properties using different precursors, as the functional groups present on the surface vary with different sources. The major drawback of green synthesized P-CDs is their potential to detect multiple ions at a time. To improve the selectivity of P-CDs towards particular metal ions, doping with different dopants(heteroatoms) can be performed to detect particular analytes. This study reveals that different functional groups present on the surface of P-CDs interact with different metal ions by forming a complex and detecting the particular metal ion. Also, the overall efficiency of the synthesis of P-CDs can be improved using different biomass (agricultural biomass, forest, and food-based biomass wastes) with different compositions of functional groups and carbon. Further, the quantum yield of P-CDs can be easily enhanced using hetero-atom-rich biowaste with some specific metal ions. In addition, the utilization of biomass-based precursors for the fabrication of P-CDs proposes a greater interest in environmental remediation and the development of sustainable technology. As we look forward, various possibilities emerge in the realm of plant-derived carbon dots (P-CDs) for heavy metal ion detection. A significant development in this field is the emergence of synthesis methods focusing on sustainability and efficiency. Researchers are looking towards novel, greener techniques for CD manufacture, which might decrease costs and environmental impacts while improving the functionality of these nanostructures. The development of multifunctional P-CDs is gaining progress day by day. These innovative techniques can incorporate sensing capabilities with additional significant qualities, including energy storage, expanding their potential uses in a variety of disciplines. Another promising trend is the incorporation of nanotechnology and smart materials. This could lead to the development of P-CDs with dynamic, real-time responses to environmental changes, delivering more precise and actionable data on heavy metal concentrations. Advanced data analytics and machine learning are becoming increasingly utilized to understand sensor outputs. The technique enhances the reliability and accuracy of the detection of heavy metal devices by enabling more complex data processing. Furthermore, this development of compact and field-deployable detection equipment is anticipated to improve accessibility and usefulness, particularly in distant or underserved areas. Finally, continued research into novel natural precursors and sustainable procedures will be critical in reducing the environmental impact of CD manufacture while increasing its effectiveness. These growing tendencies point to a future in which plant-based P-CDs play an increasingly vital role in environmental monitoring and safety.

## Figures and Tables

**Table 1:** Detection of Iron (Fe^2+^, Fe^3+^) ion greener P-CDs.

Sr. No.	Method	Natural Precursor	Detection Limit	Size of P-CDs	Linear Concentration Range	Quantum Yield (%)	Metal Ions	Reference
1.	Pyrolysis	Mangifera indica	0.62 ppm	1–5 nm		18.2%	Fe^2+^	[24]
2.	Hydrothermal	Betel leaves	0.135 μM	3.7 nm	0.3–3.3 μM	4.21%	Fe^3+^	[28]
3.	Hydrothermal	Honey	1.7 × 10^−9^ mol/L	2 nm	5.0 × 10^−9^–1.0 × 10^−4^ mol/L	19.8%	Fe^3+^	[33]
4.	Hydrothermal	Lycii Fructus	21 nM	3.3 nm	0 to 30 μM	17.2%	Fe^3+^	[34]
5.	Hydrothermal-carbonization	Chionanthus retusus	70 μM	5 ± 2 nm	0–2 μM	9%	Fe^3+^	[27]
6.	Hydrothermal	gelatin	0.2 µM	0.5–5 nm	0–50 µM	22.7%	Fe^3+^	[37]
7.	Hydrothermal	Kumquat	0.70 µM	3 nm	0–40 µM	8%	Fe^3+^	[35]
8.	Pyrolysis	Aloe-Vera extract,	33 ppb	6–8 nm	70 ppb to 100 ppm	12.37%	Fe^3+^	[40]
9.	Hydrothermal	papaya powder	0.48 μmol L^−1^	3.4 nm	1–10 μmol L^−1^	18.98%	Fe^3+^	[41]
10.	Hydrothermal	coriander leaves	0.4 µM	2.387 nm	0–60 µM	6.48%	Fe^3+^	[32]
11.	Hydrothermal	Jinhua bergamot	5.5 nM (Hg^2+^) and 0.075 μM (Fe^3+^)	50 nm	Hg^2+^ = 0.01–100 μM 0.025–100 μM for Fe^3+^	50.78%	Hg^2+^ and Fe^3+^	[12]
12.	Hydrothermal	wintersweet	Cr = 0.07 μM and Fe = 0.15 μM	9.38 nm	Cr (VI) = 0.1 to 60 μM And Fe^3+^ = 0.05 to 100 μM	14.8%	Cr^6+^ and Fe^3+^	[42]
13.	Hydrothermal	Poa Pratensis	Fe^2+^ = 1.4 and Mn^2+^ = 1.2 μM	2 nm	5.0 to 25 μM	7%	Fe^3+^ and Mn^2+^	[43]
14.	Hydrothermal	Borreria hispida	1.2 × 10^−6^ M	2 nm	-	40.8%	Fe^3+^	[44]

**Table 2:** Recognition of Mercury (Hg^2+^) ion via P-CDs.

Sr. No.	Method	Natural Precursor	Detection Limit	Size of P-CDs	Linear Concentration Range	Quantum Yield	Metal Ion	Reference
1.	Microwave	flour	0.5 nM	1–4 nm	0.0005–0.01 µM		Hg^2+^	[52]
2.	Hydrothermal	Pea	0.96 µM	20 nm	0–200 µM	12.09%	Hg^2+^	[53]
3.	Hydrothermal	Jinhua bergamot	5.5 nM (Hg^2+^) and 0.075 μM (Fe^3+^)	50 nm	Hg^2+^ = 0.01–100 μM 0.025–100 μM for Fe^3+^,	50.78%	Hg^2+^ and Fe^3+^	[12]

**Table 3:** Cu^2+^ ion recognition using greener P-CDs.

Sr. No.	Method	Natural Precursor	Detection Limit	Size of P-CDs	Linear Concentration Range	Quantum Yield	Metal Ion	Reference
1.	Pyrolysis	Eleusine coracana	10 nM	3–8 nm	0 to 100 μM	-	Cu^2+^	[63]
2.	Green	Prawn shells	5 nM	4 nm	0 to 5 µM	9%	Cu^2+^	[64]
3.	Microwave pyrolysis	Pinecone	0.005 µg/mL	15.2 nm and 42.1 nm	2.5–22.5 µg/mL	17%	Cu^2+^	[61]
4.	Hydrothermal	Bamboo leaves	115 nM	3.6 nm	0.333 to 66.6 µM	7.1%.	Cu^2+^	[60]

**Table 4:** Sensing of Lead (Pb^2+^) ion using green-precursor-derived P-CDs.

Sr. No.	Method	Precursor	Detection Limit	Size	Linear Concentration Range	Quantum Yield	Metal Ion	Reference
1.	Microwave	Potato dextrose agar	106–110 pM	4.3 nm	0–20 μM	9.0%	Pb^2+^	[76]
2.	Hydrothermal	Ginkgo biloba leaves	0.1–20.0 nM	4.18 nm	0.1–20 × 10^−3^	16.1%	Pb^2+^	[77]
3.	Acid hydrolysis	Bovine serum albumin	5.05 μM	1–2 nm	0–6 × 10^−3^ μM	-	Pb^2+^	[73]
4.	Hydrothermal	Chocolate	12.7 nM	6.41 nm	0.033 to 10 μM	-	Pb^2+^	[70]
5.	Hydrothermal	Ocimum sanctum	0.59 nM	3 nm	0.01−1.0 μM	9.3%	Pb^2+^	[71]
6.	Hydrothermal	Lantana camara berries	9.64 nM	20 nm	0–200 nM	33.15%	Pb^2+^	[75]

**Table 5:** Selective sensing of Cobalt (Co^2+^) ion using green precursor derived P-CDs.

Sr. No.	Method	Natural Precursor	Detection Limit	Size of P-CDs	Linear Concentration Range	Quantum Yield	Metal Ion	Reference
1.	Microwave	Kelp	0.39 μmol/L	3.7 nm	1–200 μmol/L	23.5%.	Co^2+^	[82]
2.	Hydrothermal	Nerium Oleander L. petals	6.45 nM	5–6 nm.	0–40 μM	3.5%	Co^2+^	[83]
3.	Hydrothermal method	Flax straw	Co^2+^ = 0.38, Cr^6+^ = 0.19 μM	2.2 nm	Co^2+^ = 0–500, Cr^6+^ = 0.5–80 μM	20.7%	Co^2+^ or Cr^6+^	[79]
4.	Microwave	Orange	1.63 μM	8.82 nm	0–200 μM	49.42%	Co^2+^	[84]

**Table 6:** Detection of Cadmium (Cd^2+^) ions using plant-derived P-CDs.

Sr. No.	Method	Precursor	Detection Limit	Size	Linear Concentration Range	Quantum Yield	Metal Ion	Reference
1.	Hydrothermal	Murraya koenigii	0.29 nM	2–8 nm	0.01–8 μM	5.4%	Cd^2+^	[86]
2.	Hydrothermal	L-arginine	0.20 μM	2.68 ± 0.67 nm	0–26.8 μM	71.6%	Cd^2+^	[89]

**Table 7:** Detection of Silver (Ag+) ions using plant-derived P-CDs.

Sr. No.	Method	Precursor	Detection Limit	Size	Linear Concentration Range	Quantum Yield	Metal Ion	Reference
1.	Hydrothermal	Broccoli	0.5 µM	2–6 nm	0 to 600 µM	-	Ag^+^	[90]
2.	Hydrothermal	Pomegranate juice	3.8 × 10^−10^ M	2–5 nm	8.3 × 10^−10^–3.3 × 10^−8^ M	-	Ag^+^	[91]
3.	Microwave	Guanine	90 nM	3.75 nm	0–80 µM	54%	Ag^+^	[93]

**Table 8:** Multi-metal-ion detection using green-precursor-derived P-CDs.

Sr. No.	Method	Precursor	Detection Limit (μM)	Size of P-CDs	Linear Concentration (μM)	Quantum Yield (%)	Metal Ions	Reference
1.	Hydrothermal	Lemon juice	32 μM	4–5 nm		21%	V^5+^	[101]
2.	Hydrothermal	Pyrus pyrifolia	0.0025 μM	2.0 ± 1.0 nm	0.005–50 μM	10.8%	Al^3+^	[96]
3.	Robust method	Camphor	-	1–4 nm		21.16%	Cd^2+^ and Hg^2+^	[49]
4.	Hydrothermal	Red beetroot	0.033 μM	5–7 nm	3 to 43 µM	27.6%	Pd^2+^	[102]
5.	Hydrothermal carbonization	Peach gum	0.64 µM	2–5 nm	0–50 µM	28.46%	Au^3+^	[99]
6.	Hydrothermal	Citric acid and diethylenetriamine	-	2.51 nm	0.558 μM	86%	Al^3+^	[98]
7.	Hydrothermal	Poa Pratensis	Fe^3+^ = 1.4 and Mn^2+^ = 1.2 μM	9 nm	5.0 to 25 μM	7%	Fe^3+^ and Mn^2+^	[43]
8.	Hydrothermal	Hibiscus Tea Waste	Ag^+^ = 0.0445 μM, Cd^2+^ = 0.1644 μM, and Cr^3+^ = 0.0546 μM	6.2 ± 0.5 nm	0–10 μM	9.2%	Ag^+^, Cd^2+^, and Cr^3+^ ions	[100]
9.	Hydrothermal carbonization	Catharanthus roseus	Al^3+^ = 0.5 μM; Fe^3+^ = 0.3 μM	5 nm	Al^3+^ = 0–6 μM	28.2%	Al^3+^ and Fe^3+^	[97]
10.	Microwave	Formamide and L-glutathione	Co^2+^ = 0.0968 μM, Fe^3+^ = 0.0617 μM, Hg^2+^ = 0.0395 μM, Pb^2+^ = 0.0371 μM	7.2 ± 1.2 nm	0.961 μM	6.49%	Co^2+^, Fe^3+^, Hg^2+^, and Pb^2+^	[106]
